# Ves-GAN: Unsupervised Vessel-Targeted Low-Dose Coronary Computed Tomography Angiography Denoising Framework

**DOI:** 10.34133/bmef.0149

**Published:** 2025-07-04

**Authors:** Xinyuan Xiang, Jiayue Li, Yan Yi, Yining Wang, Sixing Yin, Xiaohe Chen

**Affiliations:** ^1^ School of Information and Communication Engineering, Beijing University of Posts and Telecommunications, Beijing, China.; ^2^ School of Information Science and Technology, North China University of Technology, Beijing, China.; ^3^ Department of Radiology, Peking Union Medical College Hospital, Beijing, China.; ^4^ College of Artificial Intelligence, China University of Petroleum, Beijing, China.

## Abstract

**Objective:** This study aims to develop an unsupervised denoising framework for low-dose coronary computed tomography (CT) angiography (LDCTA), which reduces noise while preserving vascular structures. **Impact Statement:** This work proposes Ves-GAN, a novel denoising framework that meets the challenges of data acquisition and assumptions about noise characteristics. By providing robust noise reduction while maintaining vascular integrity, Ves-GAN facilitates more reliable clinical evaluations and improves the overall quality of cardiovascular diagnosis. **Introduction:** LDCTA minimizes radiation exposure in cardiovascular imaging but introduces noise and blurring, affecting diagnostic accuracy. Existing denoising methods, such as supervised deep learning models, require paired datasets and rely on noise assumptions. Unsupervised models show promise but often fail to preserve vascular structures, limiting clinical application. **Methods:** Ves-GAN incorporates a high-frequency-aware data augmentation strategy for robust generalization. The generator employs a high-frequency squeeze-and-excitation module to improve sensitivity to fine vascular features. Additionally, a vessel-consistency loss is introduced to preserve structural integrity during the denoising process. **Results:** On average, Ves-GAN achieves 7.5% and 10.2% improvements in peak signal-to-noise ratio and structural similarity index metrics compared to existing unsupervised models. Clinical validation involved 50 CT scans reviewed by 3 radiologists, who noted substantial enhancements in vascular clarity and lesion visibility. **Conclusion:** Ves-GAN outperforms existing unsupervised models in preserving vascular details and noise reduction, significantly enhancing clinical diagnostic reliability.

## Introduction

Coronary computed tomography (CT) angiography (CTA) is a vital diagnostic tool for cardiovascular diseases, valued for its noninvasive nature and high resolution. However, adhering to the “As Low As Reasonably Achievable” principle [1], CTA examinations are conducted with lower radiation doses and reduced contrast agent use. This often leads to increased noise and blurring, particularly in the vascular regions, which reduces vessel clarity and may result in missed diagnoses of critical conditions such as vascular stenosis and plaque occlusion [2]. Therefore, reducing noise while effectively enhancing vessels in low-dose CTA (LDCTA) images is essential for improving image quality, lesion visibility, and diagnostic precision.

To enhance the quality of LDCTA images, several reconstruction methods have become key areas of research. These methods are generally divided into 3 categories: iterative reconstruction (IR) [3], sinogram filtration-based techniques [4], and image postprocessing techniques [5]. The core principle of IR is to optimize an objective function that integrates an accurate system model, a statistical noise model, and prior knowledge about the image. While IR algorithms significantly reduce image noise, they tend to lose some fine details and are still prone to residual artifacts. Additionally, the high computational cost of IR is a major limitation, making it challenging for widespread clinical application [6]. Sinogram filtration methods, by contrast, work directly on the projection data before image reconstruction, offering a more computationally efficient solution compared to IR. However, access to sinogram data is often restricted with commercial scanners, and these techniques are also susceptible to edge blurring and resolution loss [7]. Image postprocessing techniques operate directly on the final reconstructed image itself. As a result, these techniques are unable to correct for noise or artifacts that were introduced during earlier stages of the image formation process, limiting their overall effectiveness.

Recently, deep learning (DL)-based denoising techniques have made significant strides in medical imaging, particularly in learning complex structures and noise characteristics. These techniques are generally categorized into supervised and unsupervised learning approaches. In supervised learning, DL models transform LDCTA to normal-dose images by training on paired, aligned data to reduce reconstruction loss between them [8]. Yang et al. [9] proposed a generative adversarial network (GAN) combining Wasserstein distance and perceptual similarity for CT denoising, effectively reducing noise and preserving details but requiring strictly paired images. Advanced variants like Wasserstein GAN with gradient penalty (WGAN-GP) [10] and spectral normalization for GAN (SNGAN) [11] address training instability, while perceptual similarity enhancements [9] improve detail. However, these methods face challenges including computational complexity, potential mode collapse, and, crucially, reliance on paired training data, which is difficult to obtain clinically.

Recent advances in diffusion probabilistic models offer alternatives. CoreDiff [12] uses a contextual error-modulated generalized diffusion model with LDCTA images to displace random Gaussian noise, employing a mean-preserving degradation operator to mimic CT degradation. CoCoDiff [13] implements a contextual conditional diffusion approach, training a noise estimation network to gradually convert residual images to a Gaussian distribution. While innovative, these predominantly rely on paired data. Diffusion models also present several limitations for LDCTA denoising: (a) Their strength in denoising natural image random noise is less suited for complex LDCTA noise characteristics [12]. (b) Multiple sampling steps during inference can lead to long computation times. (c) They struggle to preserve fine anatomical structures, especially small vessels critical for LDCTA, potentially losing diagnostic information. (d) They typically require large-scale datasets, which are often limited in medical imaging due to acquisition and ethical constraints.

To meet the challenge on data acquisition, various unsupervised learning methods address data acquisition challenges. BM3D [14] leverages image self-similarity for robust, training-free denoising, effective against Gaussian noise but struggles with complex non-Gaussian CT noise (especially low dose) and can compromise fine details due to predefined filtering assumptions. Among unsupervised models, self-supervised frameworks tackle paired training data scarcity. Noise-to-Noise (N2N) [15] trains on noisy image pairs of the same content, learning noise patterns effectively. However, its requirement for multiple acquisitions is often clinically impractical (increased radiation). Noise-to-Void (N2V) [16] uses a blind-spot strategy for training on individual noisy images, but its performance drops when noise and signal statistics are similar, hindering fine vascular structure preservation in coronary CTA. Noise-to-Sim (N2S) [17] improves N2V by explicitly modeling noise, but reliance on simulated noise can cause oversmoothing and difficulty distinguishing high-frequency details. Iterative denoising and reconstruction (IDR) [18] incorporates physical imaging models for better structural preservation. However, it relies on accurate forward models (errors cause artifacts) and is computationally intensive. Despite advances, existing methods often prioritize global noise reduction over preserving critical vascular structures (vital for coronary CTA), using simplified noise assumptions or lacking explicit structural consistency constraints, risking loss of clinically relevant details. Cycle-GAN [19] maps LDCTA to normal-dose images using unpaired datasets, eliminating paired data needs and avoiding explicit LDCT noise assumptions. However, for vascular imaging, Cycle-GAN’s limitations include the following: (a) prioritizing global appearance, potentially sacrificing fine vascular structures; (b) lack of direct input–output constraints leading to inconsistent feature preservation; and (c) cycle-consistency loss not specifically preserving high-frequency vascular details. Thus, the challenge persists in achieving effective global denoising while selectively enhancing vascular features and maintaining structural consistency, particularly in the context of unpaired learning scenarios that reflect real-world clinical constraints.

**Fig. 1.**
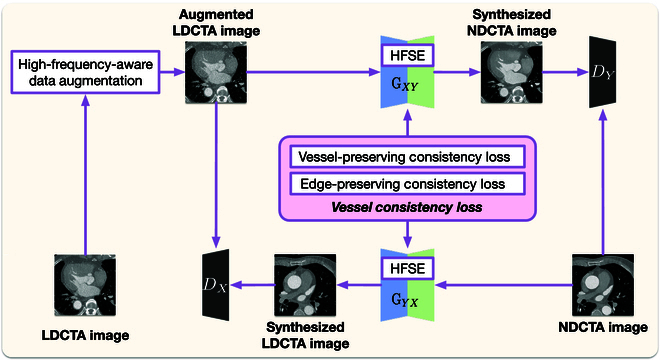
The framework of the proposed denoising method.

In order to overcome the above challenge, we propose Ves-GAN, a novel unsupervised vessel-targeted denoising framework based on the architecture of Cycle-GAN. Ves-GAN achieves global denoising of LDCTA images while selectively enhancing vascular features, maintaining structural consistency, and improving the CTA clinical diagnostic reliability. The details of our proposed framework are depicted in Fig. 1. We first incorporate a high-frequency-aware data augmentation module. This module ensures that the model generalizes well across different noisy conditions such that the robustness is improved. Once the augmented LDCTA image is obtained, we then generate synthesized NDCTA images constrained by a vessel-consistency loss. This loss is composed of a vessel-preserving consistency loss and an edge-preserving consistency loss. The vessel-preserving consistency loss encourages the generator to focus more on the quality of vascular generation such that vascular features are preserved. The generator is a segmentation model based on 2D U-Net [20]. We introduce a high-frequency squeeze-and-excitation (HFSE) module in each stage of the generator. This module enhances the generator’s sensitivity to high-frequency features by utilizing the Laplacian and Sobel operators. The edge-preserving consistency loss optimizes the generator, ensuring that the contours of the synthesized image align with those of the original, maintaining structural consistency. We test the proposed framework on a public dataset from AAPM-Mayo Clinic low-dose CT grand challenge [21] and a private clinical dataset consisting of 25,281 CT images. We conduct extensive experiments comparing our model with various state-of-the-art unsupervised models. On average, Ves-GAN achieves 7.5% and 10.2% improvements in peak signal-to-noise ratio (PSNR) and structural similarity index (SSIM) metrics compared to the existing unsupervised models. We also performed a clinical validation that involves 50 CT scans reviewed by 3 radiologists, who noted significant enhancements in vascular clarity and lesion visibility.

The remainder of this paper is organized as follows: Results presents the experimental results and performance evaluation. Discussion provides a comprehensive discussion of our findings. Materials and Methods describes the datasets, experimental setup, and proposed methodology.

## Results

### Evaluation results

In this subsection, we present the denoising performance of our model on both public and private datasets through extensive experiments. To comprehensively assess denoising effectiveness from multiple dimensions, we employ 4 complementary evaluation metrics: PSNR and SSIM to evaluate fidelity and structural similarity against reference normal-dose CT images; the no-reference Blind/Referenceless Image Spatial Quality Evaluator (BRISQUE) [22] to assess overall perceptual quality based on image statistical characteristics; and the Tenengrad function to measure image sharpness and edge clarity. Since our model is trained on unpaired data, we compare it against a variety of state-of-the-art unsupervised denoising frameworks, including BM3D, N2N, N2V, N2S, IDR, and Cycle-GAN.

#### AAPM-Mayo Clinic low-dose CT grand challenge dataset

**Fig. 2.**
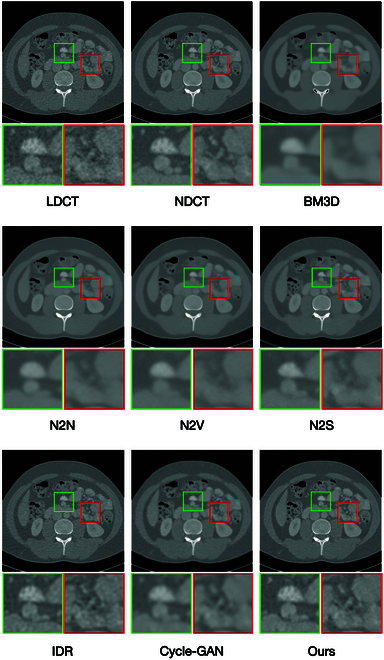
The visual results generated by a variety of unsupervised denoising models.

The quantitative and denoised imaging results are shown in Table 1 and Fig. 2, respectively. As indicated in Table 1, our model achieves superior performance across PSNR, SSIM, and BRISQUE metrics. Notably, it significantly enhances LDCTA image quality in terms of BRISQUE and Tenengrad metrics, bringing them closer to NDCTA standards. BM3D, N2N, N2V, N2S, and IDR yield considerably lower Tenengrad scores, as their tendency to oversmooth the images results in a loss of important texture details. The trends in PSNR and SSIM align with the improvements in BRISQUE and Tenengrad scores, confirming the reliability of these metrics for image quality assessment. On average, our model achieves 7.5% and 10.2% improvements in PSNR and SSIM metrics compared to the existing unsupervised models.

**Table 1.** Performance comparison of different methods

Methods	PSNR AAPM	SSIM AAPM	BRISQUE AAPM	Tenengrad AAPM	BRISQUE SPCTA-to-CTA	Tenengrad SPCTA-to-CTA
NDCT	–	–	20.43 ± 10.84	41.98 ± 6.26	22.69 ± 8.25	56.26 ± 9.96
LDCT	36.02 ± 1.54	0.86 ± 0.04	29.79 ± 9.80	49.61 ± 6.38	30.42 ± 5.35	45.43 ± 4.84
BM3D	36.17 ± 0.71	0.87 ± 0.01	40.43 ± 7.81	29.47 ± 5.03	40.47 ± 4.08	32.99 ± 8.85
N2N	33.72 ± 0.75	0.77 ± 0.04	37.11 ± 7.85	30.78 ± 5.30	37.25 ± 3.62	38.66 ± 8.85
N2V	33.99 ± 1.87	0.80 ± 0.04	37.72 ± 12.86	29.43 ± 5.67	32.62 ± 3.27	38.48 ± 7.54
N2S	36.94 ± 0.71	0.90 ± 0.01	34.81 ± 9.75	32.23 ± 5.33	27.36 ± 4.14	38.73 ± 7.62
IDR	33.67 ± 0.38	0.77 ± 0.03	41.34 ± 11.03	34.64 ± 5.64	33.24 ± 4.15	48.20 ± 7.87
Cycle-GAN	36.76 ± 1.01	0.86 ± 0.02	23.08 ± 7.77	45.50 ± 6.63	27.59 ± 5.14	49.82 ± 9.50
Ours	37.81 ± 1.04	0.91 ± 0.01	21.66 ± 6.79	43.75 ± 6.29	22.86 ± 7.43	54.16 ± 10.52

The visual results are illustrated comprehensively in Fig. 2. To facilitate detailed inspection, we use green and red boxes to highlight specific anatomical regions of interest. These annotations allow for a closer examination of the structural details and high-frequency features within the images. Upon reviewing the subfigures corresponding to BM3D, N2N, N2V, and N2S, it becomes evident that these methods result in a noticeable loss of high-frequency details, leading to a reduction in structural clarity and a less defined representation of fine anatomical features. Similarly, the results obtained from IDR and Cycle-GAN demonstrate limited efficacy in enhancing edge features or restoring image quality to a level comparable to the gold-standard NDCT images. Both methods exhibit deficiencies in preserving essential structural integrity, which is critical for accurate diagnostic evaluation. In contrast, the images generated by our proposed method exhibit significantly improved visual fidelity, closely resembling the NDCT images. This observation underscores our method’s superior capability in preserving high-frequency details and enhancing anatomical features critical for diagnostic purposes.

#### SPCTA-to-CTA dataset

**Fig. 3.**
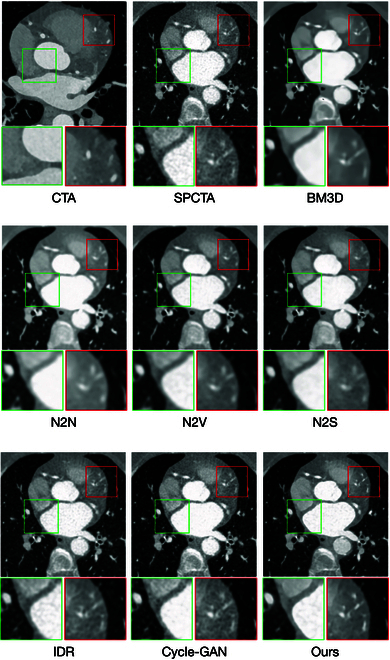
The visual results generated by a variety of unsupervised denoising models.

The quantitative and qualitative results of denoised images are comprehensively presented in Tables 1 and 2 and Fig. 3. As demonstrated in Table 1, our model consistently outperforms other unsupervised methods, achieving top-tier results in both the BRISQUE metric and the Tenengrad metric. The BRISQUE metric, which evaluates image quality without requiring a reference, reflects our model’s ability to produce images closer to the natural scene statistical norms. The Tenengrad metric, which assesses image sharpness and gradient strength, highlights our model’s superior performance in preserving structural clarity and enhancing high-frequency details critical for diagnostic interpretation.

**Table 2.** The image quality assessed by the radiologists

Methods	Vascular and edge visibility	Structural preservation	Noise reduction effectiveness	Average
CTA	5.0	5.0	5.0	5.0
SPCTA	3.0	3.0	3.0	3.0
BM3D	2.1	1.8	3.4	2.4
N2N	1.9	1.9	3.6	2.4
N2V	2.3	2.0	3.5	2.6
N2S	2.3	1.9	4.2	2.8
IDR	3.1	3.6	3.1	3.2
Cycle GAN	3.6	3.8	3.2	3.5
Ours	4.8	4.9	4.6	4.7

Figure 3 offers visual validation of our model’s capability in effectively suppressing random noise while retaining essential high-frequency details. Specific areas of interest, such as vascular features and high-frequency edge regions, are marked with green and red boxes, respectively, for closer inspection. In these marked regions, our model demonstrates its strength in maintaining vascular integrity and structural consistency. Unlike BM3D and N2N, which often introduce oversmoothing, our model avoids the compromise of texture and edge clarity. While BM3D and N2N produce cleaner images, they fail to distinctly represent blood vessel contours and organ edges, critical for accurate diagnostics.

Alternative methods such as N2V, N2S, IDR, and Cycle-GAN show some capacity to preserve high-frequency features, yet they lack the ability to enhance tissue details or emphasize critical structures effectively. In stark contrast, our model leverages a combination of advanced mechanisms, including the vessel-preserving consistency loss, edge-preserving consistency loss, and the HFSE module integrated into the generator. This unique architecture not only excels in noise reduction but also ensures the preservation and enhancement of high-frequency anatomical details. The synergy between these components enables our model to produce images with sharper blood vessel morphology, more defined organ edges, and enhanced tissue structure representation. These improvements are evident in the visual results, where our model delivers images that closely resemble the reference standard, underscoring its superior capability in balancing noise suppression with structural preservation. The clarity of vascular features and the prominence of organ contours in the denoised images illustrate the practical advantages of our approach for clinical diagnostics.

Additionally, we conduct an independent real-world test to evaluate the impact of different denoising models on radiologists’ diagnostic accuracy and interpretative experience. This study focuses on how various denoising methods affect the visibility and clarity of vessels and the contours of core organs in CTA images.

To ensure objectivity, reliability, and reproducibility of the scoring process, we implement several methodological safeguards. First, a standardized scoring strategy is developed, which contains detailed criteria for each score level (1 to 5) across all evaluation aspects. Second, all testing images are randomized and presented in a consistent viewing environment with standardized display settings and ambient conditions. Third, each radiologist performs the evaluations independently in separate sessions to prevent influence from other readers.

As shown in Table 2, in terms of vascular and edge visibility—a critical metric for coronary CTA diagnostic value—our method achieved a mean score of 4.8 (out of 5), approaching the reference standard of normal-dose CTA (5.0) and substantially outperforming the next best competitor, Cycle-GAN (3.6). This pronounced improvement in vascular visualization directly impacts diagnostic confidence in vascular stenosis analysis and detection. For structural preservation, our method again demonstrated near-reference performance (4.9 vs. 5.0 for normal-dose CTA), maintaining the integrity of anatomical structures while substantially surpassing other techniques. Notably, traditional methods like BM3D (1.8) and other self-supervised approaches (N2N, N2V, and N2S) performed particularly poorly in this category (scores of 1.9 to 2.0), suggesting their tendency to oversmooth critical anatomical features. Regarding noise reduction effectiveness, our approach (4.6) significantly outperformed all competitors while maintaining essential structural details. While N2S achieved reasonable noise suppression (4.2), this came at the cost of structural and vascular detail preservation, as evidenced by its lower scores in those categories (2.3 and 1.9 respectively). The consistent superiority of our method across all clinical evaluation metrics validates its effectiveness in addressing the core challenge of LDCTA denoising: achieving substantial noise reduction while preserving critical diagnostic features. The radiologists’ assessments align with our quantitative technical metrics, confirming that the improvements in BRISQUE and Tenengrad translate to clinically meaningful enhancements in image quality and diagnostic value.

### Ablation study

We conduct an ablation study to evaluate the performance of our model under different configurations.

#### Effectiveness of skip connections

To analyze the impact of various skip connection modules in the generator’s U-shaped architecture, we replace the original skip connections with spatial and channel squeeze and excitation (scSE) [23], self-attention (SA) [24], convolutional block attention module (CBAM) [25], and our proposed HFSE module. The results, shown in Table 3, highlight the effectiveness of different designs in enhancing denoising performance and structural fidelity based on BRISQUE and Tenengrad metrics.

**Table 3.** Performance comparison of different methods

		BRISQUE	Tenengrad
Skip connection modules	Identity mapping	23.70 ± 7.50	53.29 ± 10.75
	scSE	26.41 ± 7.92	51.63 ± 10.05
	Self-attention	24.01 ± 6.37	50.18 ± 8.14
	CBAM	23.10 ± 8.71	53.35 ± 9.12
Loss combinations	Original	27.59 ± 5.14	49.82 ± 9.50
	Original + edge	23.67 ± 7.18	51.34 ± 9.42
	Original + vessel	23.47 ± 7.20	51.09 ± 9.30
Augmentation strategy	No data augmentation	25.94 ± 5.91	52.41 ± 9.14
	Vessel and edge blurring	23.69 ± 8.04	53.96 ± 10.69
	Gaussian blurring	22.54 ± 8.36	50.77 ± 10.07
	Poisson blurring	24.40 ± 7.32	52.43 ± 9.11
	Salt-and-pepper blurring	24.66 ± 6.11	52.52 ± 9.72
LDCT(SPCTA)		30.42 ± 5.35	45.43 ± 4.84
NDCT(CTA)		22.69 ± 8.25	56.26 ± 9.96
Ours		22.86 ± 7.43	54.16 ± 10.52

The scSE and SA modules offer limited improvements compared to the original skip connections. However, CBAM and our HFSE module demonstrate significant enhancements, with HFSE outperforming CBAM in retaining high-frequency details and edge clarity. This confirms the HFSE module’s effectiveness in denoising and preserving structural consistency, providing strong experimental evidence for its advantages in handling unpaired LDCTA data.

#### Impact of vessel-consistency loss

To assess the effectiveness of vessel-consistency loss, we compare different combinations of loss functions, all trained and tested on the same dataset:

1. Original Cycle-GAN loss: Includes adversarial loss, cycle consistency loss, and identity loss.

2. With edge-preserving consistency loss: Adds edge-preserving consistency loss to the original Cycle-GAN loss.

3. With vessel-preserving consistency loss: Adds vessel-preserving consistency loss to the original Cycle-GAN loss.

As shown in Table 3, incorporating either edge-preserving or vessel-preserving loss improves performance over the original loss. While edge-preserving loss enhances contour sharpness and boundary clarity, vessel-preserving loss excels in preserving subtle vascular features. Combining both losses results in synergistic improvements in denoising quality, edge clarity, and vascular detail preservation. This comprehensive enhancement improves reconstructed image quality and clinical applicability.

From Table 3, it is evident that incorporating either the edge-preserving loss or the vessel-preserving loss into the original Cycle-GAN loss function enhances denoising performance on reference-free metrics. While the quantitative results show no significant difference between the models utilizing edge-preserving consistency loss and vessel-preserving consistency loss, the visual comparisons in Figs. 2 and 3 highlight their distinct contributions. The edge-preserving consistency loss enhances the sharpness of contours, ensuring clear and well-defined boundaries while avoiding oversmoothing. On the other hand, the vessel-preserving consistency loss excels in maintaining subtle vascular features, improving the visibility and resolution of vessels in the denoised images. When both losses are combined, the model achieves a synergistic effect, leading to notable improvements in overall denoising quality, edge clarity, and vascular detail preservation. This comprehensive enhancement significantly elevates the quality of reconstructed images and their clinical applicability.

#### The effectiveness of high-frequency-aware data augmentation

To investigate the effectiveness of the proposed high-frequency-aware data augmentation strategy, a variety of data augmentation strategies are tested. These strategies include the following:

1. No data augmentation: Training with the original LDCTA images without any data augmentation strategy.

2. Vessel and edge blurring: Randomly apply one of Gaussian filtering, median filtering, or mean filtering to the vessel and edge regions of LDCTA images to simulate the loss of image details.

3. Gaussian blurring: Adding Gaussian noise to the entire LDCTA images.

4. Poisson blurring: Adding Poisson noise to the entire LDCTA images.

5. Salt-and-pepper blurring: Adding salt-and-pepper noise to the entire LDCTA images.

The results in Table 3 show that all of these data augmentation methods have improved BRISQUE scores, with the strategy of Gaussian blurring showing the most significant enhancement in BRISQUE. However, this strategy exhibited a declining trend in Tenengrad scores. This sharply contrasts with the results of vessel and edge blurring: although vessel and edge blurring resulted in relatively limited improvements in BRISQUE, it significantly enhanced Tenengrad performance. This phenomenon indicates that the personalized vessel and edge blurring can effectively unlock the model’s potential in subtle structures and edge sharpening, thereby achieving better performance in retaining high-frequency details and sharpening.

## Discussion

Based on our comprehensive evaluation, several observations require a deeper discussion to understand the advantages of our proposed model.

### Fluctuation on evaluation metrics

According to Table 1, within the column “PSNR AAPM”, we also found out that Ves-GAN has higher fluctuation than other methods, excluding N2V. A similar observation can be found for the BRISQUE and Tenengrad evaluations on the single-phase CTA (SPCTA)-to-CTA dataset. The reason behind the higher fluctuation can be explained from 2 aspects. As demonstrated in the “Augmentation strategy” part of Table 3, it indicates that while models are trained without data augmentation, they show minimal fluctuation, but also achieve significantly lower performance on average. The enhanced data augmentation approach we implemented introduces some variability but substantially improves overall performance metrics, effectively compensating for the increased fluctuation. This trade-off between fluctuation and performance is a deliberate design choice, as the improved robustness and generalization capabilities outweigh the minor inconsistencies in results. The other aspect that leads to the higher fluctuation focuses on the model structure. Within Table 3, the results shown in “Skip connection modules” and “Loss combinations” reveal that these architectural components also introduce some variability. However, the considerable performance gains they provide justify this trade-off. Specifically, our HFSE module in skip connections and the vessel-consistency loss enhance the model’s ability to preserve vascular details, which is essential for clinical applications even if it comes with slightly higher variation in quantitative metrics.

### Clinical relevance and radiologist assessment

Perhaps the most compelling evidence for our method’s effectiveness comes from the radiologist evaluation. The near-reference performance in vascular and edge visibility (4.8/5.0) and structural preservation (4.9/5.0) demonstrates that our approach produces images that closely approximate normal-dose CTA quality. This is particularly significant given that these aspects directly impact diagnostic confidence in vascular stenosis analysis—the primary clinical application of coronary CTA. The striking gap between our method’s scores and those of competing approaches (with the next best competitor, Cycle-GAN, scoring only 3.6 for vascular visibility) underscores the clinical significance of our technical innovations. Traditional methods performed particularly poorly in structural preservation (scores of 1.8 to 2.0), highlighting a fundamental limitation in their application to vessel-rich imaging contexts.

### Mechanism analysis

The superior performance of our approach can be attributed to the synergistic effect of 3 key components: the vessel-preserving consistency loss, edge-preserving consistency loss, and the HFSE module. While conventional denoising methods treat all image regions equally, our approach specifically prioritizes the preservation and enhancement of vascular structures through these specialized mechanisms. The HFSE module’s effectiveness is particularly evident in the enhanced Tenengrad scores, which measure edge sharpness—a critical factor for vessel visualization. Meanwhile, our consistency losses ensure that the structural integrity of the image is maintained throughout the denoising process, addressing a key limitation of existing GAN-based approaches.

## Materials and Methods

### Dataset

Ves-GAN is tested on a public dataset from AAPM-Mayo Clinic low-dose CT grand challenge and an SPCTA-to-CTA self-collected private clinical dataset. The pre-trained vascular segmentation model and edge-extraction model are trained on another public dataset ImageCAS [26].

#### AAPM-Mayo Clinic low-dose CT grand challenge dataset

This public dataset contains 10 cases of clinical data, and the total number of the samples is 2,378. Each case includes paired full-dose and quarter-dose (25%) synthetic LDCT images, both acquired at a resolution of 512 × 512 pixels. For the Mayo Clinic low-dose CT grand challenge dataset (AAPM dataset), the definition of normal dose and low dose is clearly established in the dataset specifications. The normal-dose images were acquired at 120 kV and 200 quality reference mAs (QRM), while the low-dose images were simulated at 120 kV and 50 QRM, representing a quarter of the standard dose (25% dose ratio). All scans were acquired using a Siemens SOMATOM Flash scanner in the portal venous phase. In our experiments, we utilized the 3-mm-thick images with B30 reconstruction kernel from this dataset. In our study, LDCT images are used as the target, while the corresponding LDCT images are input into Ves-GAN for training. For data preparation, 2 cases are randomly selected as the test set, while the remaining 8 cases are used as the training set.

#### SPCTA-to-CTA dataset

In addition to a public dataset, we also test the proposed denoising framework on a self-collected private dataset. SPCTA and CTA both use an 80-kV tube voltage and 300-mAs tube current, with a scan range of approximately 12 to 15 cm, but they differ significantly in data acquisition methods and radiation dose. SPCTA is a single-phase image extracted from ATP-stress dynamic myocardial CTP data. CTP is acquired using a prospectively ECG-triggered table shuttle mode, with a total dose-length product (DLP) of approximately 400 mGy·cm. Since CTP consists of 10 phases, the DLP for a single phase is approximately 40 mGy·cm. Based on the formula for effective radiation dose (ED), where ED = DLP×*k*, with *k* = 0.014 mSv/mGy·cm, the estimated effective radiation dose for SPCTA is approximately 0.56 mSv. In contrast, CTA employs a prospectively ECG-triggered high-pitch spiral acquisition mode. Based on the scan parameters and a CT dose index volume of approximately 14 mGy, the calculated DLP is approximately 200 mGy·cm, resulting in an effective radiation dose of 2.8 mSv. All CTA and CTP images were reconstructed using a Bv36 medium sharp convolution kernel with an ADMIRE level 3 IR algorithm, a slice thickness of 0.6 mm, and an increment of 0.4 mm. CTP images were analyzed using dedicated postprocessing software (syngo.via VB10 myocardial perfusion, Siemens), and multiphase CTA images from CTP scans were loaded into a 4-dimensional (4D) imaging analysis program (syngo.via VB10, dynamic angio) for SPCTA extraction. Motion correction was performed for all images, and a multi-frequency band filter was applied for 4D noise reduction in cases without significant motion artifacts. Additionally, a region of interest was placed in the ascending aorta to evaluate the time attenuation curve, with the peak phase selected as SPCTA. Transverse, 2-chamber short-axis, 2-chamber, and 4-chamber long-axis images were subsequently reformatted. In summary, the radiation dose of SPCTA is significantly lower than that of CTA (0.56 mSv versus 2.8 mSv), accounting for only one-fifth of CTA (a difference of approximately 5 times), and the 2 methods differ in data acquisition techniques, image postprocessing, and imaging objectives.

The clinical significance of our model in this experimental setup is introduced as follows. Standard CT perfusion plus CTA scans incur high radiation, time, and costs. SPCTA, reconstructing angiography from perfusion data to avoid separate CTA, has limited adoption due to poor image quality, especially in vascular detail visualization [27]. Our Ves-GAN framework addresses this by using SPCTA as low-quality input and CTA as high-quality target, emphasizing vascular enhancement for coronary artery evaluation. By improving SPCTA image quality, Ves-GAN can potentially eliminate dedicated CTA scans, reducing patient radiation, examination time, contrast volume, and healthcare costs, while enhancing diagnostic confidence through better vessel clarity and preservation. This demonstrates how our DL innovations translate to improved patient care, radiation safety, and healthcare resource utilization. The dataset comprises 8,131 CTA (NDCTA, 27 patients) and 17,150 SPCTA (LDCTA, 70 patients) 512×512 chest slices. We designate 1,500 SPCTA slices (8 patients) as the test set; the remaining SPCTA and all CTA data are for training. This unpaired dataset from diverse patients offers a realistic LDCTA-to-NDCTA conversion scenario.

#### ImageCAS dataset

This dataset is designed for high-precision coronary artery segmentation tasks. It includes coronary CT images from multiple patients with detailed coronary annotations and high-quality image data. In our study, the 2 pre-trained models are trained on this dataset. The vascular segmentation network focuses on accurately delineating coronary artery structures, while the edge segmentation network utilizes binary edge maps generated by the Canny algorithm [28] as labels to capture subtle edge information in the images.

### Experimental setup

In the experimental setup, the PyTorch framework version 1.12.1 was utilized. All experiments were conducted on NVIDIA A6000 GPUs and Intel i9-12900K CPUs during both training and testing phases. When training the dataset, each LDCTA image of size 512 × 512 was scaled down to 286 × 286 and then randomly cropped to 256 × 256 for data augmentation; the corresponding NDCTA images were also processed in the same manner. Additionally, we only performed high-frequency-aware data augmentation on the LDCTA images, applying blur to the vessels or edges within the LDCTA and with a 30% chance of adding random noise to the entire image. The types of noise included Gaussian noise, Poisson noise, and salt-and-pepper noise. To ensure model convergence, we conducted 1,000 training epochs and set the weights for the adversarial loss, cycle consistency loss, identity loss, vessel preservation loss, and edge preservation loss to 1, 10, 5, 1, and 1, respectively. During training, network parameters were optimized by minimizing the composite loss function using the Adam optimizer, with the learning rate gradually decreasing from 2 × 10^−4^ to 1 × 10^−6^ following a cosine annealing schedule. In the inference process, the 512 × 512 LDCTA images were cropped into overlapping patches to evaluate the network’s performance.

### Evaluation metrics

In this study, we select 4 evaluation metrics—PSNR, SSIM, BRISQUE, and Tenengrad function—to comprehensively assess the performance of the denoising algorithm from multiple dimensions. These 4 metrics complement each other, offsetting their respective strengths and limitations to ensure that the evaluation of denoising effectiveness is both comprehensive and reliable.

PSNR and SSIM, as gold standards for reference-based image quality assessment, accurately evaluate the similarity and structural fidelity between the denoised images and the NDCT images. In contrast, for reference-free image quality assessment methods, BRISQUE, a reference-free image quality assessment method, analyzes image statistical characteristics. Originally designed based on statistical regularities in natural images (e.g., specific distributions of locally normalized luminance coefficients), its direct applicability to CT images warrants careful consideration. CT images exhibit distinct noise characteristics (quantum noise, beam hardening artifacts, and reconstruction-dependent variations) that may deviate from BRISQUE’s underlying statistical assumptions. Despite these potential differences, we empirically validated BRISQUE’s performance on CT images using the paired AAPM dataset. Calculating both reference-based metrics (PSNR and SSIM) and BRISQUE scores for denoised CTA images, we found a strong correlation between them. This empirical validation suggests that, while its underlying statistical assumptions may differ, BRISQUE can effectively capture image quality variations in the CT domain. The core idea of BRISQUE is to utilize natural scene statistical features to describe the local structure and texture distribution of an image, and then model these features using a support vector machine to predict a quantitative quality score for the given image. The lower the score, the closer the image is to a natural and high-quality state; the higher the score, the more significant distortions such as noise, blur, or artifacts are present in the image.

Tenengrad function assesses the sharpness of the images by detecting the clarity of image edges. Its fundamental concept is to measure the sharpness and clarity of an image by detecting the intensity of high-frequency components such as edges and textures within the image. The Tenengrad function first uses the Sobel operator to extract horizontal and vertical gradients from the image, and then calculates the statistics of the gradient magnitudes, using these as a clarity score. The higher the Tenengrad score, the greater the sharpness of the image and the clearer the image contours. This is because a higher Tenengrad score indicates more pronounced high-frequency components, such as sharper edges and more defined textures, resulting in a clearer and more detailed image. This score is calculated as follows:

$$\frac{1}{N}\sum_{i,j}\sqrt{{\left(G_{h}\left(i,\;j\right)\right)}^{2}+{\left(G_{v}\left(i,\;j\right)\right)}^{2}},$$ (1)

where $G_{h}\left(i,j\right)$ and $G_{v}\left(i,j\right)$ respectively represent the horizontal and vertical gradient values at pixel $\left(i,j\right)$ of the image, which are calculated using the Sobel operator.

To validate the effectiveness of using BRISQUE and Tenengrad on a reference-free dataset, we first conduct comparative experiments on the paired AAPM dataset [21]. The experimental results demonstrate that BRISQUE and Tenengrad exhibited a highly consistent trend with PSNR and SSIM in evaluating the denoising performance. This confirms that these 2 reference-free metrics remain reliable even in the absence of paired data. Consequently, in the SPCTA-to-CTA dataset, we employ BRISQUE and Tenengrad to evaluate the denoising performance, ensuring the algorithm’s applicability and effectiveness across different data environments. This multi-metric evaluation strategy not only enhances the credibility of the research findings but also provides robust theoretical support for the clinical application of the denoising algorithm.

Beyond these objective quantitative evaluations, to further assess the practical clinical utility and perceptual image quality improvements from an expert perspective, a qualitative evaluation was conducted. The test involves 3 radiologists, all of whom are chief radiologists from Peking Union Medical College Hospital with over 10 years of clinical experience in radiological imaging diagnosis. This ensures that the study results maintain high professional standards and clinical relevance. To minimize bias, all radiologists remain blinded to the type of denoising model used during the evaluation process. Each radiologist is required to perform comparative assessments of 50 pairs of images before and after denoising, with the evaluation aspects mainly including the following:

• Vascular and edge visibility: The clarity and prominence of vessels and their edges.

• Structural preservation: The integrity and clarity of anatomical structures and core organs.

• Noise reduction effectiveness: The degree to which noise has been suppressed in the image.

### Methods

#### Unsupervised denoising model

To meet the challenge of obtaining paired CTA images and avoid assumptions about LDCT noise characteristics, we propose Ves-GAN, which is based on the architecture of Cycle-GAN [19]. In contrast to Cycle-GAN, Ves-GAN achieves global denoising while enhancing vascular features and maintaining structural consistency.

**Fig. 4.**
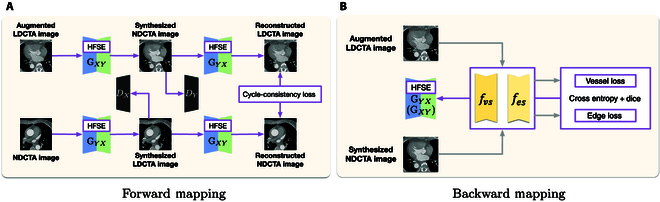
The denoising procedure based on unpaired LDCTA and NDCTA images. (A) The procedure on forward mapping. (B) The procedure on backward mapping.

As depicted in Fig. 4, in the forward mapping, the 2 generators, $G_{\mathrm{XY}}$ and $G_{\mathrm{YX}}$, learn the mappings from LDCTA to NDCTA and from NDCTA to LDCTA, respectively. *X* and *Y* represent the LDCTA image domain and the NDCTA image domain, respectively. Each generator utilizes an encoder–decoder architecture based on 2D U-Net [20], where the encoder extracts semantic features of vascular structures and organs from the LDCTA domain, while the decoder transforms these features into the NDCTA domain. Although 2D U-Net is originally designed for segmentation, we adapt the U-Net architecture for cross-domain image translation, emphasizing preservation of anatomical structures, particularly vascular details, during the domain transformation process. We propose to incorporate an HFSE module in each stage of the generator. This module enhances the generator’s sensitivity to high-frequency features by utilizing the Laplacian and Sobel operators. The discriminators $D_{X}$ and $D_{Y}$ play a crucial role in the adversarial training process by evaluating the realism of the synthesized images in *X* and *Y*. The goal of denoising is achieved by translating LDCTA images into NDCTA images through the $G_{\mathrm{XY}}$ generator (translating NDCTA images into LDCTA images through the $G_{\mathrm{YX}}$).

To globally denoise the LDCTA image while enhancing image diversity by selectively blurring vascular features and high-frequency edges, we first introduce a high-frequency-aware data augmentation module. This module applies localized filtering techniques and adds various types of noise to LDCTA images. By intentionally blurring the vessels and high-frequency edges, the localized filtering increases the variability of the training data, making the model’s denoising capability for vascular and high-frequency regions more generalized. Additionally, introducing different types of noise during the denoising task ensures that the model generalizes well across diverse noisy conditions, thereby improving its robustness. After obtaining the augmented LDCTA image, we generate synthesized NDCTA images constrained by the vessel-consistency loss. This loss comprises a vessel-preserving consistency loss and an edge-preserving consistency loss, which enforce explicit vessel enhancement and preserve structural details.

#### High-frequency-aware data augmentation module

The process of high-frequency-aware data augmentation contains 3 steps. The first step is the execution of a vessel segmentation model and an edge-extraction model, where both models are based on 2 pre-trained Res-UNet models [29]. The architecture of the 2 pre-trained models are the same, which is described in Table 4. The 2 pre-trained models $f_{v}$ and $f_{e}$, respectively segment vascular structures and high-frequency components along edges, generating binary masks $S_{v}$ and $S_{e}$ for these regions, ensuring that subsequent processing focuses on the associated features. The 2 masks are then combined to form a unified structural mask: $S=S_{v}\cup S_{e}$.

**Table 4.** The architecture of the 2 pre-trained Res-UNet models

	ResNet encoder	U-Net decoder
	Layer	Layer
	(conv, channel, stride, padding)	(conv, channel, stride, padding)
	Output size	Output size
Stage 0	Conv: 7 × 7, 64, 2, 3	Interpolate: 2
	64 × 128 × 128	Conv: 3 × 3, 32, 1, 1
		Conv: 3 × 3, 32, 1, 1
		Conv: 3 × 3, 2, 1, 1
		2 × 256 × 256
Stage 1	Maxpool: 3 × 3, stride = 2	Interpolate: 2
	Conv: 3 × 3, 64, 1, 1	Cat: 128 + 64
	Conv: 3 × 3, 64, 1, 1	Conv: 3 × 3, 64, 1, 1
	64 × 64 × 64	Conv: 3 × 3, 64, 1, 1
		64 × 128 × 128
Stage 2	Conv: 3 × 3, 128, 2, 1	Interpolate: 2
	Conv: 3 × 3, 128, 1, 1	Cat: 256 + 64
	128 × 32 × 32	Conv: 3 × 3, 128, 1, 1
		Conv: 3 × 3, 128, 1, 1
		128 × 64 × 64
Stage 3	Conv: 3 × 3, 256, 2, 1	Interpolate: 2
	Conv: 3 × 3, 256, 1, 1	Cat: 256 + 128
	256 × 16 × 16	Conv: 3 × 3, 256, 1, 1
		Conv: 3 × 3, 256, 1, 1
		256 × 32 × 32
Activation	LeakyReLU	ReLU
Params	7.06M	

The second step subjects to blur the *S* with localized Gaussian, mean, and median filtering techniques. The blurring process is defined as:

$$\begin{array}{c} I_{\text{blur}}\left(i,j\right)=\left\{\begin{array}{cc} B\left(I_{\text{LDCT}}\left(i,\;j\right)\right), & \left(i,\;j\right)\in S\\ \;I_{\text{LDCT}}\left(i,\;j\right), & \left(i,\;j\right)\notin S \end{array}\right. \end{array}$$ (2)

where $B\left(\cdot \right)$ is randomly selected as one of Gaussian filtering, mean filtering, or median filtering, and $\left(i,\;j\right)$ denotes a pixel position in the image.

In the third step, random noise *N* with a probability *p* is added to the blurred image $I_{\text{blur}}$. This process augments the LDCTA image by enhancing critical structures, while introducing realistic noise for robustness in downstream tasks. The noise type includes Gaussian, Poisson, or salt-and-pepper noise. By learning to process images with various noise profiles, the denoising model can better differentiate high-frequency features from noises, enhancing their ability to extract vascular features.

For Gaussian noise:

$$I_{\mathrm{gs}}\left(i,\;j\right)=I_{\text{blur}}\left(i,\;j\right)+n\left(i,\;j\right),$$ (3)

where $n\left(i,\;j\right)∼N\left(0,\;\sigma^{2}\right)$, and σ is the standard deviation.

For Poisson noise:

$$I_{\mathrm{po}}\left(i,\;j\right)∼\text{Poisson}\left(I_{\text{blur}}\left(i,\;j\right)\right)$$ (4)

For salt-and-pepper noise:

$$I_{\mathrm{sp}}\left(i,\;j\right)=\left\{\begin{array}{cc} 0, & 0\leq r\left(i,\;j\right)<k_{1}\\ \;255, & k_{1}\leq r\left(i,\;j\right)<k_{2}\\ I_{\text{blur}}\left(i,\;j\right), & k_{2}\leq r\left(i,\;j\right)<1 \end{array}\right.$$ (5)

where $r\left(i,\;j\right)$ is a random number uniformly distributed in [0, 1], and $k_{1},k_{2}$ are constants. In summary, the augmented LDCTA image $I_{\mathrm{aug}}\left(i,\;j\right)$ can be described as:

$$I_{\mathrm{aug}}\left(i,\;j\right)=\left(1-q\right)\cdot I_{\text{blur}}+q\cdot I_{n}\left(i,\;j\right)$$ (6)

where $I_{n}\left(i,\;j\right)\in \left[I_{\mathrm{gs}}\left(i,\;j\right),I_{\mathrm{po}}\left(i,\;j\right),I_{\mathrm{sp}}\left(i,\;j\right)\right]$, and $q\in \left[0,1\right]$, with the probability of *q* = 1 being *p*.

**Fig. 5.**
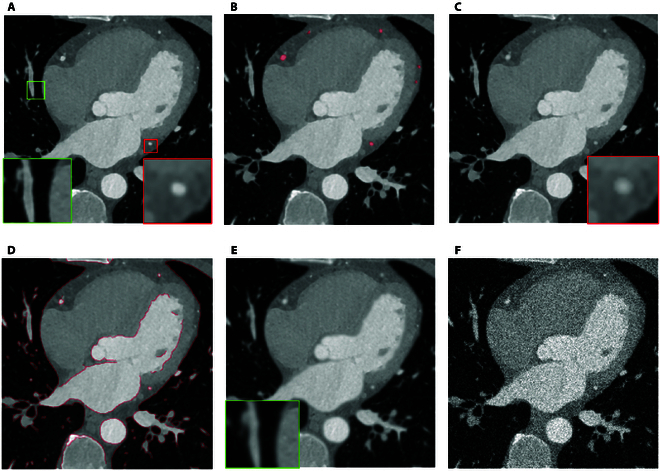
(A) The original image. (B) The segmentation output of vascular features. (C) The effect after filtering techniques (vessel). (D) The segmentation output of high-frequency features. (E) The effect after filtering techniques (edges). (F) Augmented image.

Figure 5 displays an example image processed by the high-frequency-aware data augmentation module. Figure 5A shows an original image, where we have marked the high-frequency edge regions and the areas corresponding to vascular structures with green and red boxes, respectively. The features within these boxes have been magnified for closer inspection. Figure 5B and D show the segmented outputs of vascular structures and high-frequency edge regions in red, respectively. Figure 5C and E illustrate the effects of localized Gaussian, mean, and median filtering, creating a more blurred appearance in these highlighted areas compared to the original image’s enlarged sections. Figure 5F demonstrates the application of Gaussian, Poisson, and salt-and-pepper noise, producing a noisier image than the original one in Fig. 5A, with some vascular structures becoming obscured due to the added noises.

In clinical practice, the ability to preserve both vessel and edge details is crucial for accurate cardiovascular diagnosis. Fine vascular details are necessary for detecting anomalies such as stenosis, while sharp edges help radiologists accurately assess vessel boundaries and surrounding tissues. Recognizing that the mixed nature and inherent variability of clinical datasets can introduce biases and significantly impede a model’s ability to generalize effectively across different data sources, we sought robust mechanisms to address this challenge. Drawing inspiration from strategies for learning dataset-invariant features and enhancing model generalization explored in [30,31], we introduced the vessel-consistency loss. This loss is specifically designed to guide our model to focus on fundamental and stable vascular characteristics that are inherently consistent across diverse patient populations, imaging equipment, and acquisition protocols. By promoting the learning of these genuinely representative and invariant features, the vessel-consistency loss aims not only to improve the model’s generalization performance on varied datasets but also to mitigate potential biases that could arise from learning spurious, dataset-specific correlations. By incorporating this vessel-consistency loss, the proposed framework significantly enhances the diagnostic value of LDCTA images, ultimately supporting improved patient outcomes and more effective cardiovascular assessments.

The vessel-consistency loss is a fundamental component in the denoising framework, specifically designed to ensure the preservation of vascular structures and the overall integrity of LDCTA images during denoising. Denoising tasks often compromise fine vascular details, which are critical for clinical diagnosis and assessment. To mitigate this, the vessel-consistency loss integrates 2 essential components: vessel-preserving consistency loss and edge-preserving consistency loss. These elements work together to constrain the generators, ensuring that the synthesized denoised images retain the vascular features and structural outlines of the original LDCTA images.

The vessel-preserving consistency loss primarily addresses the challenge of maintaining vascular fidelity during the denoising process. Vascular structures, such as coronary arteries, are characterized by fine and intricate details that can be easily distorted or blurred by traditional denoising methods. This component ensures that the generated NDCTA images retain the vascular features of the original LDCTA images. The original LDCTA image is first subjected to the high-frequency-aware data augmentation module to enhance data diversity. The augmented LDCTA image is then fed into the generator $G_{\mathrm{XY}}$ to produce an NDCTA image. This generator is tasked with reducing noise while preserving anatomical features. However, without explicit constraints, the generator may inadvertently smooth out vascular structures. To counter this, a pre-trained vascular segmentation model is employed to extract the vascular mask from the generated NDCTA image. This segmentation process identifies and highlights the vascular regions within the image.

Simultaneously, the vascular segmentation logits (soft output of the segmentation model) from the original LDCTA image serve as the label. By comparing the vascular mask from the denoised image to that of the original image, the vessel-preserving consistency loss is computed. This loss penalizes discrepancies between the 2 segmentations, guiding the generator to maintain vascular consistency. The calculation process is described as follows:

$$\begin{array}{c} \begin{array}{l} L_{\mathrm{vp}}\left(G_{\mathrm{XY}},G_{\mathrm{YX}}\right)=E_{x∼p\left(x\right)}\left[∥f_{\mathrm{vs}}\left(x\right)-f_{\mathrm{vs}}\left(G_{\mathrm{XY}}\left(I_{\mathrm{aug}}\left(x\right)\right)\right){∥}_{1}\right]\\ \;+E_{y∼p\left(y\right)}\left[∥f_{\mathrm{vs}}\left(y\right)-f_{\mathrm{vs}}\left(G_{\mathrm{YX}}\left(y\right)\right){∥}_{1}\right], \end{array} \end{array}$$ (7)

where $I_{\mathrm{aug}}\left(x\right)$ is the output image generated by the high-frequency-aware data augmentation module. $f_{\mathrm{vs}}$ denotes the vascular segmentation network. *x* and *y* are the samples of LDCTA and NDCTA images, respectively.

The vessel-preserving consistency mechanism encourages the model to prioritize vascular features, preventing the attenuation or loss of crucial anatomical structures. This ensures that the fine details of the vasculature are preserved throughout the denoising pipeline, directly enhancing the accuracy and reliability of clinical assessments based on these images.

In addition to preserving vascular features, it is equally important to maintain the overall structural integrity of the image. One of the major limitations of generative models, such as Cycle-GAN, is the potential for structural inconsistencies, particularly in edge regions. Blurring or distortion of edges can compromise the delineation of vessels and other anatomical structures, reducing the diagnostic value of the denoised images.

The edge-preserving consistency loss is introduced to address this issue by reinforcing the alignment of edges between the original and synthesized images. This ensures that not only the vascular regions but also the surrounding anatomical structures are preserved without distortion. The process starts by extracting contours and edges from the original LDCTA image using a pre-trained edge-extraction model. These edges are used as labels representing the high-frequency structural boundaries within the image. Next, the augmented LDCTA image is processed by the generator $G_{\mathrm{XY}}$ to produce the NDCTA image. The edges of the NDCTA image are then extracted using the same edge-extraction model. By comparing the edges of the NDCTA with those from the original LDCTA, the edge-preserving consistency loss is computed. This loss penalizes misalignment or deformation in the edges, forcing the generator to produce denoised images that maintain the same structural outlines as the original images. The calculation process is as follows:

$$\begin{array}{c} \begin{array}{l} L_{\mathrm{ep}}\left(G_{\mathrm{XY}},G_{\mathrm{YX}}\right)=E_{x∼p\left(x\right)}\left[∥f_{\mathrm{es}}\left(x\right)-f_{\mathrm{es}}\left(G_{\mathrm{XY}}\left(I_{\mathrm{aug}}\left(x\right)\right)\right){∥}_{1}\right]\\ \;+E_{y∼p\left(y\right)}\left[∥f_{\mathrm{es}}\left(y\right)-f_{\mathrm{es}}\left(G_{\mathrm{YX}}\left(y\right)\right){∥}_{1}\right], \end{array} \end{array}$$ (8)

where $f_{\mathrm{es}}$ represents the edge extraction network.

The edge-preserving consistency loss plays a crucial role in preventing structural deformations that often occur during denoising, ensuring that key boundaries and contours remain intact. This is essential for maintaining the visual clarity and diagnostic accuracy of the denoised images, as clear edges are vital for identifying vessel boundaries, stenosis, and lesions.

#### Network architecture

**Fig. 6.**
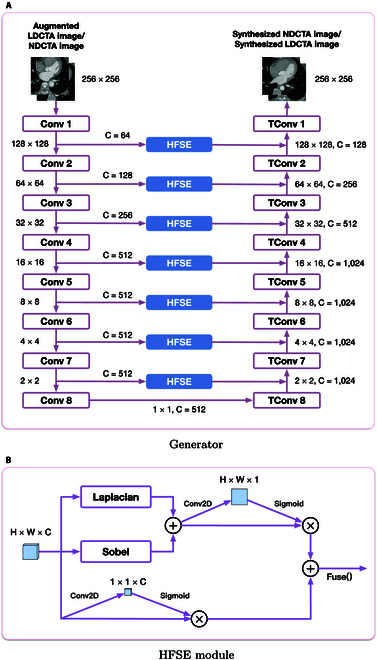
The architecture of the generator and the HFSE module.

Ves-GAN includes 2 generators, $G_{\mathrm{XY}}$ and $G_{\mathrm{YX}}$, and 2 discriminators, $D_{X}$ and $D_{Y}$. We adopt a U-Net architecture for the 2 generators, replacing the original 3 × 3 convolutions and transposed convolutions with 4 × 4, all with a stride of 2. The reason behind this setup is explained as follows. At first, inspired by Wu et al. [32], the 4 × 4 convolution kernels provide a larger receptive field than the standard 3 × 3 kernels, enabling the model to capture broader spatial context and enhancing its ability to perceive the overall contours in the images. Second, the combination of 4 × 4 kernels with stride 2 effectively integrates downsampling (or upsampling) operations, resulting in fewer network layers and improved training stability by mitigating gradient vanishing problems. This configuration also helps avoid checkerboard artifacts [33] by ensuring that the kernel size is divisible by the stride length. Third, the larger convolution kernels complement our 70 × 70 PatchGAN discriminator architecture, promoting better feature matching between the generator and discriminator, which ultimately enhances the fine detail quality in the synthesized images. Inspired by Chen et al. [34], the network depth is also increased to improve performance. Furthermore, as shown in Fig. 6A, we replace the original skip connections in U-Net with an HFSE module to enhance the generator’s sensitivity to high-frequency features. For the 2 discriminators $D_{X}$ and $D_{Y}$, we use a 70 × 70 PatchGAN [35] to capture fine-grained features of the image, improving the detail quality of synthesized images.

The architecture of the HFSE module is depicted in Fig. 6B. We first employ the Laplacian and Sobel operators to extract high-frequency information, with the kernels defined as follows:

$$\begin{array}{c} \;K_{\mathrm{lap}}=\left[\begin{array}{ccc} -1 & -1 & -1\\ -1 & 8 & -1\\ -1 & -1 & -1 \end{array}\right],\;K_{\text{sobel}\_i}=\left[\begin{array}{ccc} -1 & 0 & 1\\ -2 & 0 & 2\\ -1 & 0 & 1 \end{array}\right],\\ K_{\text{sobel}\_j}=\left[\begin{array}{ccc} -1 & -2 & -1\\ 0 & 0 & 0\\ 1 & 2 & 1 \end{array}\right]. \end{array}$$ (9)

The Laplacian operator and the Sobel operator are commonly used edge detection tools. The Laplacian operator is a second-order derivative filter that highlights areas with rapid intensity changes, enabling it to effectively detect fine edges and local structural details. In contrast, the Sobel operator emphasizes edge information by calculating the gradient of image intensities. High-frequency information is then obtained by convolving the input features *F* with the above kernels, which are calculated as:

$$F_{\text{sobel}}=\sqrt{{\left(F_{\text{sobel}\_i}\right)}^{2}+{\left(F_{\text{sobel}\_j}\right)}^{2}},F_{\text{combined}}=F_{\mathrm{lap}}+F_{\text{sobel}},$$ (10)

where $F_{\text{sobel}\_i}=F^{*}K_{\text{sobel}\_i}$, $F_{\text{sobel}\_j}=F^{*}K_{\text{sobel}\_j}$, and $F_{\mathrm{lap}}=$$F^{*}K_{\mathrm{lap}}$.

By convolving the input LDCT image features *F* with Laplacian and Sobel kernels, high-frequency information within the feature maps is effectively captured. This process enables the model to focus more on the details and edges of the images, which is particularly important for preserving blood vessels and other fine structures in medical images.

Then, the spatial attention mechanism and channel attention mechanism are applied to highlight important features. The spatial attention mechanism primarily focuses on the spatial dimensions of the image, identifying which regions are more important for the current task. Under the constraints of vascular preservation loss and edge preservation loss, the network can automatically concentrate on these crucial areas through the spatial attention mechanism. This enhances the denoising effect without sacrificing key details. On the other hand, the channel attention mechanism concentrates on the channel dimensions of the feature maps, identifying which feature channels are more important for the current task. Different channels may correspond to different features; for example, some channels might specialize in edge information, while others focus on texture information. By utilizing the channel attention mechanism, the network can dynamically adjust the weights of each channel, enhancing the response to important features and further improving the denoising performance. Next, a feature fusion module is used to integrate this information, as follows:

$$F^{\prime }=M_{\text{fusion}}\left(A_{S}\left(F_{\text{combined}}\right)+A_{C}\left(F\right)\right).$$ (11)

The feature fusion module is designed to integrate features that have been processed by the spatial attention and channel attention mechanisms. Specifically, the fusion module combines feature information from different sources to create a richer and more comprehensive feature representation. This step ensures that, during the denoising process, the network considers both the spatial structure of the image and the significance of each feature channel. Consequently, it achieves more efficient feature utilization and information transmission.

Finally, we introduce a learnable parameter α to control an adaptive residual connection, combining the original input with the fused features:

$$F_{\text{output}}=F+\alpha \cdot F^{\prime }.$$ (12)

Adaptive residual connections are a method of combining the original input with the processed features. In this approach, a learnable parameter α is introduced to dynamically adjust the weights of these 2 components. Specifically, the network can automatically determine the extent to which it retains information from the original input and the extent to which it relies on the fused high-frequency compensation features based on the requirements of the current task. This adaptive mechanism allows the network to maintain the overall structure of the image while enhancing its ability to recover details and edges.

This design allows the network to adaptively balance the original features and high-frequency compensation features, enhancing the ability to restore fine structures while effectively suppressing noise, thereby improving the quality of the denoised images. By explicitly extracting and compensating for high-frequency components and incorporating attention mechanisms, the HFSE significantly enhances the generator’s ability to retain critical details in medical images.

#### Training loss

The training loss of Ves-GAN includes another 3 types of losses, which are adversarial loss, cycle-consistency loss, and identity loss.

Adversarial loss is a fundamental component of Cycle-GAN, derived from the framework of GANs. Its main objective is to encourage the generator to produce realistic noise-free images, fooling the discriminator into being unable to distinguish between synthesized and real images. In the image denoising task, the generator converts noisy images into clean images, while the discriminator is responsible for distinguishing between synthesized noise-free images and real noise-free images. The adversarial loss is as follows:

$$\begin{array}{c} L_{\mathrm{adv}}\left(G_{\mathrm{XY}},G_{\mathrm{YX}},D_{X},D_{Y}\right)=L_{\mathrm{adv}}^{X}\left(G_{\mathrm{XY}},D_{Y},x,y\right)\\ \;+L_{\mathrm{adv}}^{Y}\left(G_{\mathrm{YX}},D_{X},x,y\right), \end{array}$$ (13)

where

$$\begin{array}{c} \begin{array}{l} L_{\mathrm{adv}}^{X}\left(G_{\mathrm{XY}},D_{Y}\right)=E_{y∼p\left(y\right)}\left[\text{log}\;D_{Y}\left(y\right)\right]\\ \;-E_{x∼p\left(x\right)}\left[\text{log}\;D_{Y}\left(G_{\mathrm{XY}}\left(x\right)\right)\right], \end{array} \end{array}$$ (14)

$$\begin{array}{c} \begin{array}{l} L_{\mathrm{adv}}^{Y}\left(G_{\mathrm{YX}},D_{X}\right)=E_{x∼p\left(x\right)}\left[\text{log}\;D_{X}\left(x\right)\right]\\ \;-E_{y∼p\left(y\right)}\left[\text{log}\;D_{X}\left(G_{\mathrm{YX}}\left(y\right)\right)\right]. \end{array} \end{array}$$ (15)

Relying solely on adversarial loss may cause the generator to learn arbitrary mappings, failing to ensure reasonable and consistent transformations. To address this, Cycle-GAN introduces cycle consistency loss, ensuring that the original image can be recovered through bidirectional transformations by the 2 generators:

$$\begin{array}{c} \begin{array}{l} L_{\mathrm{cyc}}\left(G_{\mathrm{XY}},G_{\mathrm{YX}}\right)=E_{x∼p\left(x\right)}\left[∥x-G_{\mathrm{YX}}\left(G_{\mathrm{XY}}\left(x\right)\right){∥}_{1}\right]\\ \;+E_{y∼p\left(y\right)}\left[∥y-G_{\mathrm{XY}}\left(G_{\mathrm{YX}}\left(y\right)\right){∥}_{1}\right]. \end{array} \end{array}$$ (16)

Identity loss ensures that the generator does not make unnecessary changes to images that are already in the target domain, thereby preserving their original structure and color information:

$$\begin{array}{c} \begin{array}{l} L_{\mathrm{id}}\left(G_{\mathrm{XY}},G_{\mathrm{YX}}\right)=E_{x∼p\left(x\right)}\left[∥x-G_{\mathrm{YX}}\left(x\right){∥}_{1}\right]\\ \;+E_{y∼p\left(y\right)}\left[∥y-G_{\mathrm{XY}}\left(y\right){∥}_{1}\right]. \end{array} \end{array}$$ (17)

Based on the descriptions of the above losses, the training loss of the proposed denoising model is:

$$\begin{array}{c} \begin{array}{c} \begin{array}{c} \begin{array}{ll} & L\left(G_{\mathrm{XY}},G_{\mathrm{YX}},D_{X},D_{Y}\right)=L_{\mathrm{adv}}\left(G_{\mathrm{XY}},G_{\mathrm{YX}},D_{X},D_{Y}\right)+L_{\mathrm{cyc}}\left(G_{\mathrm{XY}},G_{\mathrm{YX}}\right)\\ & +L_{\mathrm{id}}\left(G_{\mathrm{XY}},G_{\mathrm{YX}}\right)+L_{\mathrm{vp}}\left(G_{\mathrm{XY}},G_{\mathrm{YX}}\right)+L_{\mathrm{ep}}\left(G_{\mathrm{XY}},G_{\mathrm{YX}}\right). \end{array} \end{array} \end{array} \end{array}$$ (18)

The network is optimized through alternating training:

$$\underset{D_{X},D_{Y}}{\text{max}}\underset{G_{\mathrm{XY}},G_{\mathrm{YX}}}{\text{min}}L\left(G_{\mathrm{XY}},G_{\mathrm{YX}},D_{X},D_{Y}\right)$$ (19)

## Data Availability

Data supporting the results demonstrated by this study are available within the main text. The ImageCAS Dataset used for training the vessel and edge extraction networks is publicly available at: https://www.kaggle.com/datasets/xiaoweixumedicalai/imagecas/. For denoising experiments, we utilized 2 datasets: the publicly available AAPM dataset, which can be accessed at: https://www.cancerimagingarchive.net/collection/lctsc/, and our in-house SPCTA-to-CTA dataset. The SPCTA-to-CTA dataset was obtained from our institution’s private database under appropriate ethics approval for the current study and therefore cannot be made publicly available due to patient privacy restrictions.
